# Pretreatment of the Antagonistic Yeast, *Debaryomyces hansenii*, With Mannitol and Sorbitol Improves Stress Tolerance and Biocontrol Efficacy

**DOI:** 10.3389/fmicb.2020.00601

**Published:** 2020-04-15

**Authors:** Xiaobing Ming, Yong Wang, Yuan Sui

**Affiliations:** ^1^Department of Plant Pathology, Agriculture College, Guizhou University, Guiyang, China; ^2^Chongqing Key Laboratory of Economic Plant Biotechnology, College of Landscape Architecture and Life Science/Institute of Special Plants, Chongqing University of Arts and Sciences, Chongqing, China

**Keywords:** antagonistic yeast, biocontrol efficacy, mannitol, sorbitol, stress tolerance

## Abstract

The effect of exogenous mannitol and sorbitol on the viability of the antagonist yeast, *Debaryomyces hansenii*, when exposed to oxidative and high-temperature stress was determined. Results indicated that both the 0.1 M mannitol (MT) and 0.1 M sorbitol (ST) treatments improved the tolerance of *D. hansenii* to subsequent oxidative and high-temperature stress. MT or ST cells had a significantly higher level of cell survival, elevated the gene expression of *catalase 1* (*CAT1*) and *copper-zinc superoxide dismutase* (*SOD1*), as well as the corresponding enzyme activity. Treated cells also exhibited a lower accumulation of intracellular reactive oxygen species (ROS), and a higher content of intracellular mannitol and sorbitol relative to non-treated, control yeast cells, when exposed to a subsequent oxidative (30 mM H_2_O_2_) or heat (40.5°C) stress for 30 min. Additionally, MT and ST yeast exhibited a higher growth rate in kiwifruit wounds, and a greater ability to inhibit postharvest blue mold (*Penicillium expansum*) and gray mold (*Botrytis cinerea*) infections. The present study indicates that increased antioxidant response induced by mannitol and sorbitol in *D. hansenii* can enhance stress tolerance and biocontrol performance.

## Introduction

Postharvest fungal decay of fruits reduces their availability and economic return. While the use of synthetic, chemical fungicides remain the main method of controlling fungal decay organisms, the use of antagonistic yeasts as biological control agents has been comprehensively explored over the last three decades (Liu et al., 2013, 2018; Droby et al., 2016; Usall et al., 2016; Wisniewski et al., 2016; Carmona-Hernandez et al., 2019; Ferraz et al., 2019). Among the yeasts identified as potential biocontrol agents, *Debaryomyces hansenii* has been reported to be effective during storage against several different postharvest decay fungi in a variety of fruits, including apple (Czarnecka et al., 2019), grapefruit (Droby et al., 1989), lime (Hernández-Montiel et al., 2010), muskmelon (Rivas-Garcia et al., 2019) and papaya (Hernandez-Montiel et al., 2018).

Once antagonistic yeasts have been applied to harvested commodities, a range of factors, such as temperature, oxidative stress, solute stress, and pH can influence their viability. Concomitantly, the level of stress tolerance of antagonistic yeasts is closely related to their ability to survive and proliferate on and in host tissues, as well as to their biocontrol efficacy against pathogens (Castoria et al., 2003; Macarisin et al., 2010; Sui et al., 2015; Spadaro and Droby, 2016). Therefore, enhancing yeast stress tolerance may represent a useful strategy for improving the efficacy of antagonistic yeasts (Abadias et al., 2001; Liu et al., 2012; Wang et al., 2018).

Sugar alcohols from a wide range of sources, including sorbitol and mannitol, are widely used for food, pharmaceutical, and other industrial applications. Mannitol, a six-carbon, non-cyclic sugar alcohol, is the most abundant polyol in nature, occurring in bacteria, fungi, algae, lichens, and in at least 70 species of vascular plants. Various functional roles have been postulated for mannitol in these organisms, including carbon storage, free radical scavenging, osmoregulation, and acting as a compatible solute (Seckin et al., 2009). Mannitol has been reported to play an important role in the osmotic protection of celery (Pharr et al., 1995). Mannitol transport and intracellular metabolism is a major aspect of the response of olive trees to osmotic stress (Conde et al., 2011). Sorbitol also plays an important role in the regulation of osmotic pressure in cells. A growth medium amended with sorbitol induces an adaptive osmotic response in nematodes (Chandler-Brown et al., 2015). Sorbitol in pear trees also plays an important role in osmotic adjustments in pear leaves (Larher et al., 2009). Shen et al. (1999) proposed that the accumulation of mannitol and sorbitol in the model yeast, *Saccharomyces cerevisiae*, may have dual functions, namely, facilitating osmotic adjustment and also contributing to the maintenance of redox homeostasis. Teixidó et al. (1998) improved the ecological fitness and environmental stress tolerance of the biocontrol yeast, *Candida sake*, by altering the concentration of intracellular sugar alcohols (glycerol, erythritol, arabitol, and mannitol) and sugars. To the best of our knowledge, however, there is little information about the effect of sugar alcohols on the antioxidant response of antagonistic yeast in the biological control of postharvest diseases.

The objective of the present study was to determine the effect of sugar alcohol (mannitol or sorbitol) on the antioxidant response and subsequent stress tolerance and biocontrol efficacy of the biocontrol yeast, *D. hansenii.* More specifically, the study determined (1) cell viability of mannitol-treated (MT) or sorbitol-treated (ST) cells to a subsequent exposure to oxidative (H_2_O_2_) and high-temperature stress; (2) the effect of mannitol or sorbitol treatment on the expression of antioxidant genes, *catalase 1* (*CAT1*) and *copper-zinc superoxide dismutase* (*SOD1*), and their corresponding enzyme activity; (3) the intracellular accumulation of reactive oxygen species (ROS); (4) the growth of the yeast in kiwifruit wounds; and (5) the biocontrol efficacy *D. hansenii* yeast cells pretreated with sorbitol or mannitol against the infection of kiwifruit by blue mold (*Penicillium expansum*) and gray mold (*Botrytis cinerea*).

## Materials and Methods

### Antagonistic Yeast

The antagonistic yeast, *D. hansenii* M13, was originally isolated from the surface of mango fruit and identified by its general morphology and DNA sequence of the ITS region of ribosomal DNA (Leaw et al., 2006). It was cultured in a yeast-peptone-dextrose (YPD) broth (10 g of yeast extract, 20 g of peptone, and 20 g of dextrose in 1 L of water). Twenty milliliters of YPD was placed in a 50-mL conical flask and inoculated with *D. hansenii* at an initial concentration of 10^5^ cells/mL determined using a hemocytometer. Yeast cultures were incubated at 25°C on a rotary shaker at 200 r.p.m. for 16 h.

### Fungal Pathogens

The fungal pathogens, *B. cinerea* and *P. expansum*, were isolated from infected fruit and maintained on potato dextrose agar (PDA) at 4°C. To reactivate the culture and verify its pathogenicity, the pathogens were inoculated into wounds of kiwifruit fruit and re-isolated onto PDA once the infection was established. Spore suspensions of the two pathogens were obtained from 2-week-old PDA cultures incubated at 25°C. The spore number was calculated with a hemocytometer, and the concentration was adjusted to 1 × 10^4^ spores/mL with sterile distilled water.

### Fruit

Kiwifruit (*Actinidia deliciosa* cv. Hayward) were harvested at commercial maturity. The average quality parameters at the time of harvest were: 9.8 Brix, 67 N firmness, and 96 g fresh weight per fruit. Fruits without wounds or rot were selected based on uniformity of size, disinfected with 2% (v/v) sodium hypochlorite for 2 min, rinsed with tap water, and air-dried. These fruits were used in the biocontrol assay.

### Mannitol and Sorbitol Treatment of *D. hansenii*

Yeast cultures were grown overnight and the culture tubes were then centrifuged at 8,000 × *g* for 3 min. The yeast cells were subsequently washed three times with sterile distilled water to remove any residual medium, centrifuging the cells between each wash (Liu et al., 2012). Washed cells were resuspended in the same volume (20 mL) of fresh YPD, supplemented with mannitol or sorbitol at a final concentration of 0.1 M and incubated at 25°C for 2 h on a rotary shaker at 200 rpm. The selected concentration of mannitol and sorbitol was based on preliminary experiments. Control cells were subjected to the same process, but in a medium that was not supplemented with mannitol or sorbitol. Cells were harvested by centrifugation at 8,000 × *g* for 3 min and washed three times with sterile distilled water in order to remove any residual medium. The mannitol-treated (MT), sorbitol-treated (ST) and non-treated (NT) control yeast samples were suspended in water at 1 × 10^7^ cells/mL and used in the subsequent analyses.

### Effect of Mannitol and Sorbitol on Stress Tolerance of *D. hansenii*

The effect of polyol pretreatment on tolerance of *D. hansenii* to oxidative and high-temperature stress was measured as previously described (Deveau et al., 2010), with slight modification. To measure oxidative stress tolerance, a 10 mL sample of MT, ST, or NT (control) yeast at a concentration of 1 × 10^7^ cells/mL was placed in a 50-mL conical flask and exposed to oxidative stress conditions, 30 mM H_2_O_2_, at 25°C for 30 min on a rotary shaker at 200 rpm. To assay high temperature tolerance, 1 mL of MT, ST, or NT (control) yeast cells (1 × 10^7^ cells/mL) was placed into several 1.5 mL Eppendorf tubes. The tubes were then placed in a 40.5°C water bath for 30 min, and manually shaken once every 5 min. At designated time points, 50 μL of serial 10-fold dilutions of the samples were spread on YPD agar plates. The plates were incubated at 25°C for 3 days, and then the number of Colony-Forming Units (CFUs) per plate was determined. Survival rates were expressed as a percentage of the number of colonies produced after the oxidative or high-temperature stress treatments relative to the number of CFUs produced from samples prior to each treatment (Liu et al., 2012). Three replicates were used for each treatment, and each experiment was repeated three times.

### Measurement of Intracellular ROS

The oxidant-sensitive probe, 2,7-dichlorodihydrofluorescein diacetate (H_2_DCFDA; Invitrogen, Eugene, OR, United States), was used to assess the intracellular accumulation of ROS in yeast cells (Liu et al., 2011). Yeast cell samples were collected from cultures exposed to 30 mM H_2_O_2_ or 40.5°C for 30 min. The cell samples taken prior to exposure to the oxidative or heat stress served as time 0. Yeast cell samples were washed with phosphate buffered saline (PBS) buffer (pH 7.0) and resuspended in the same buffer containing 25 μM H_2_DCFDA (dissolved in dimethyl sulfoxide). The suspension was incubated in the dark at 30°C for 1 h. After washing twice with PBS buffer, spores were examined under a FV3000 confocal microscope (Olympus, Tokyo, Japan) equipped with a UV-light source using a 488 nm excitation and 510 nm emission filter combination. Ten fields of view from each slide (at least 200 cells) were randomly chosen and the number of cells producing visible levels of ROS was counted. The ROS level was calculated as a percentage (number of fluorescing cells divided by number of cells present in the bright field image × 100). Three biological replicates were used for each sample in each assay, and the experiments were repeated three times.

### RNA Isolation and RT-qPCR Analysis of Gene Expression

Total RNA from yeast samples (MT, ST, and NT) was extracted, treated with DNase, and purified using an Ultrapure RNA Kit (CWBIO, Beijing, China) according to the manufacturer’s instructions. RNA quality was evaluated by gel electrophoresis and spectrophotometric analysis (Waltham, MA, United States). First-strand cDNA was synthesized using a TransScript One-Step gDNA Removal and cDNA Synthesis SuperMix kit (TransGen Biotech, Beijing, China). The resulting cDNA was used for RT-qPCR analysis following the manufacturer’s protocol. Briefly, each RT-qPCR reaction was carried out in a 20 μL reaction including 10 μL of GoTaq qPCR Master Mix (Promega, Madison, Wisconsin, United States) and 200 nmol of each gene-specific primer. The RT-qPCR was conducted on a qTOWER 2.2 (Analytik Jena AG, Germany) using the following cycling conditions: 95°C for 30 s; 40 cycles of 95°C for 5 s followed by 58°C for 15 s, 72°C for 10 s. The expression level of two target genes, *CAT1* and *SOD1*, was analyzed using gene-specific primers (Table 1). Transcript levels of *18S rRNA* gene served as an internal control. The 2^–ΔΔCT^ method was used to calculate relative expression (Livak and Schmittgen, 2001). To ensure that single products were amplified, each PCR reaction was subjected to a melting curve analysis of the amplification products. PCR products were cloned and sequenced to verify their identity. There were three biological replicates and three technical replicates for each treatment, and the experiment was repeated three times.

**TABLE 1:** Gene-specific primers used in RT-qPCR analysis of gene expression.

**Gene name Size (bp)**	**NCBI accession No.**	**Primer sequence**	**Annealing temperature (°C)**	**Product size (bp)**
*CAT1*	XM460824	F:TTCTCCGACCGTGGTACTCC R: GCCTGCTTCTTCGTTGGTCA	58	156
*SOD1*	AF327448	F: GGTGGCGGTGTTAAGAGGTG R: ACGGGTTGAAGTGAGGTCCA	58	191
*18S rRNA* [Frame2]	NG063361	F: AATTGACGGAAGGGCACCAC R: TAAGAACGGCCATGCACCAC	58	146

### Assay of Antioxidant Enzyme Activity

Yeast cell samples were collected from cultures exposed to 40.5°C or 30 mM H_2_O_2_ for 30 min. Samples of yeast cells taken prior to exposure to the oxidative or heat stress served as time 0. Yeast cells in the collected samples were disrupted in liquid nitrogen and suspended in chilled potassium phosphate buffer (0.1 M, pH 7.4). The cell homogenate was centrifuged at 10,000 × *g* for 20 min at 4°C and the supernatant was used for the enzyme assays. The activity of catalase (CAT) and superoxide dismutase (SOD) was measured using a commercial assay kit (Nanjing Jiancheng Bioengineering Institute, Nanjing, China), and expressed as U per mg protein. Protein content was determined using the Bradford assay with bovine serum albumin used to construct a standard curve (Bradford, 1976). One unit of CAT activity was defined as the decomposition of 1 μmol H_2_O_2_ per second in the reaction system, while one unit of SOD activity was defined as the amount of enzyme causing 50% inhibition in the nitro blue tetrazolium (NBT) reduction (Reverberi et al., 2005; Liu et al., 2011). Three biological replicates were used for each sample in each assay, and the experiments were repeated three times.

### Determination of Intracellular Mannitol and Sorbitol

Intracellular mannitol and sorbitol were extracted as described in a previous study (Hua et al., 2015) with slight modification. Yeast cell samples were collected by centrifugation at 8,000 × *g* for 3 min and resuspended in HPLC-grade water. The cell samples were then disrupted with the use of a Q125 sonicator (QSonica, Newtown, CT, United States) for 2 min, boiled for 5 min, and then cooled to room temperature. After vortexing, the mixture in each tube was centrifuged at 1,840 × *g* for 10 min. The supernatant was filtered through a 0.2 μm filter membrane before determination of mannitol and sorbitol levels using an HPLC equipped with a carbohydrate analysis column and a refractive index detector (Agilent Series 1200, Agilent Technologies, Santa Clara, CA, United States). For mannitol, the mobile phase was acetonitrile-water (75:25 in volume) at 1 mL/min (Liu et al., 2009). For sorbitol, the mobile phase was acetonitrile-water (80:20 in volume) at 0.8 mL/min (Hua et al., 2015). Mannitol or sorbitol was quantified using a standard (Sigma-Aldrich, Shanghai, China) with a linear response range of 0.05–10 mg mL^–1^. Intracellular mannitol and sorbitol concentrations were presented as μmol per gram fresh weight of yeast cells (Abadias et al., 2000). Three biological replicates were used for each sample in each assay and the experiments were repeated three times.

### Population Dynamics of *D. hansenii* in Wounds of Kiwifruit Fruits

Three wounds (4 mm deep × 3 mm wide) were made on the equator of each kiwifruit with a sterile nail, and a 10 μL suspension of MT or ST and NT *D. hansenii* cells (1 × 10^7^ cells/mL) was administered to each wound to characterize the population dynamics of *D. hansenii* in wounds of kiwifruit fruit. Fruit samples were collected one hour after their initial application to the wounds and then daily for a period of 4 days. Yeast populations were measured as described by Liu et al. (2012). Briefly, yeasts were recovered by removing ten samples of wounded tissues with a cork borer (1 cm diameter × 1 cm deep). Samples were then ground with a mortar and pestle in 10 mL sterile distilled water. Subsequently, 50 μL of serial 10-fold dilutions were spread on YPD agar plates. Samples taken at 1 h after treatment served as time 0. Fruits stored at 25°C were assessed each day for 4 days. Colonies were counted after incubation of YPD agar plates at 25°C for 2 days and expressed as the Log10 CFU per wound. There were three biological replicates evaluated for each treatment, and the experiment was repeated three times.

### Biocontrol Assay

The determination of biocontrol efficacy was determined as described by Wang et al. (2018). Three wounds (4 mm deep × 3 mm wide) were made on the equator of each fruit with a sterile nail. A 10 μL suspension of NT, MT, or ST cells of *D. hansenii* (1 × 10^7^ cells/mL) was administered to each wound. Sterile distilled water served as a control. After fruits were air-dried for 2 h, 10 μL of either *P. expansum* or *B. cinerea* suspension (1 × 10^4^ spores/mL) were inoculated into each wound. Treated fruits were placed in a covered plastic food tray. Each tray was enclosed with a polyethylene bag in order to maintain high humidity (approximately 95% RH) and stored at 25°C. Disease incidence and lesion diameter of kiwifruit fruits were recorded after 4 days. Each treatment contained three biological replicates with ten fruits per replicate and the experiment was repeated three times.

### Data Analysis

All statistical analyses were performed using SPSS version 20.0 (SPSS Inc., United States) software. Data with a single variable (treatment) were analyzed by a one-way ANOVA, and mean separations were performed using a Duncan’s multiple range test. Differences at *P* < 0.05 were considered significant. Data presented in this paper were pooled across three independent repeated experiments.

## Results

### Effect of Mannitol and Sorbitol on Stress Tolerance of *D. hansenii*

**FIGURE 1:**
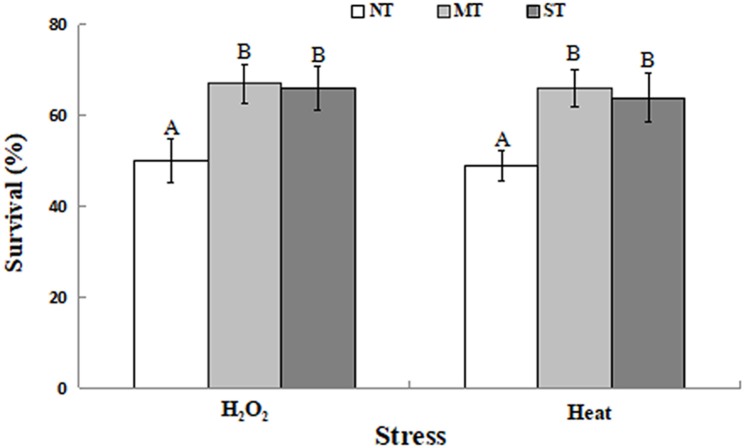
Percent viability of non-treated (NT) control, mannitol-treated (MT), and sorbitol-treated (ST) *D. hansenii* cells subjected to a subsequent oxidative (30 mM H_2_O_2_), or heat (40.5°C) stress for 30 min. Data represent the mean ± standard deviation of three independent experiments, where each experiment consisted of three biological replicates (*n* = 9). Columns with different letters are significantly different according to a Duncan’s multiple range test at *p* < 0.05.

Mannitol-treated (MT) or sorbitol-treated (ST) yeast cells exhibited significantly higher viability than non-treated (NT) cells. As presented in Figure 1, the viability of NT yeast cells was about 50%, after exposure to 30 mM H_2_O_2_ or 40.5°C for 30 min. In contrast, the viability of MT or ST yeast cells was significantly higher, after exposure to 30 mM H_2_O_2_ or 40.5°C for 30 min, respectively. These results demonstrate that yeast cells pretreated with mannitol or sorbitol had a statistically significant higher level of viability compared to NT cells.

### Effect of Mannitol and Sorbitol on Intracellular Accumulation of ROS in *D. hansenii*

**FIGURE 2:**
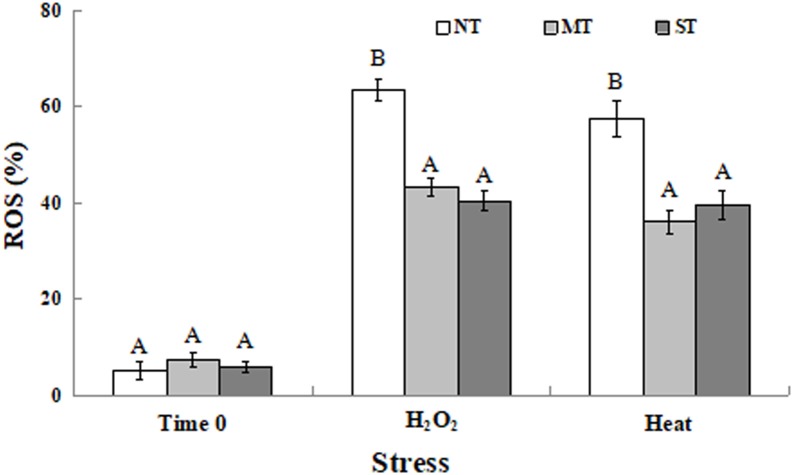
Percentage of non-treated (NT) control, mannitol-treated (MT), and sorbitol-treated (ST) *D. hansenii* cells exhibiting ROS accumulation subjected to a subsequent oxidative (30 mM H_2_O_2_), or heat (40.5°C) stress for 30 min, at time 0 (prior to exposure to the subsequent oxidative or heat stress). Data represent the mean ± standard deviation of three independent experiments, where each experiment consisted of three biological replicates (*n* = 9). Columns with different letters are significantly different according to a Duncan’s multiple range test at *p* < 0.05.

At time 0 (the time point after the 2-hour pretreatment but prior to exposure to the oxidative or high-temperature stress), the percentage of MT and ST cells, as well as NT cells, exhibiting ROS was less than 10% (Figure 2). This percentage increased when cells were exposed to either a 30-minute treatment of 30 mM H_2_O_2_ or 40.5°C. Both MT and ST cells exhibited a significantly lower percentage of cells exhibiting ROS compared to NT cells under each specific stress.

### Antioxidant Gene Expression

**FIGURE 3:**
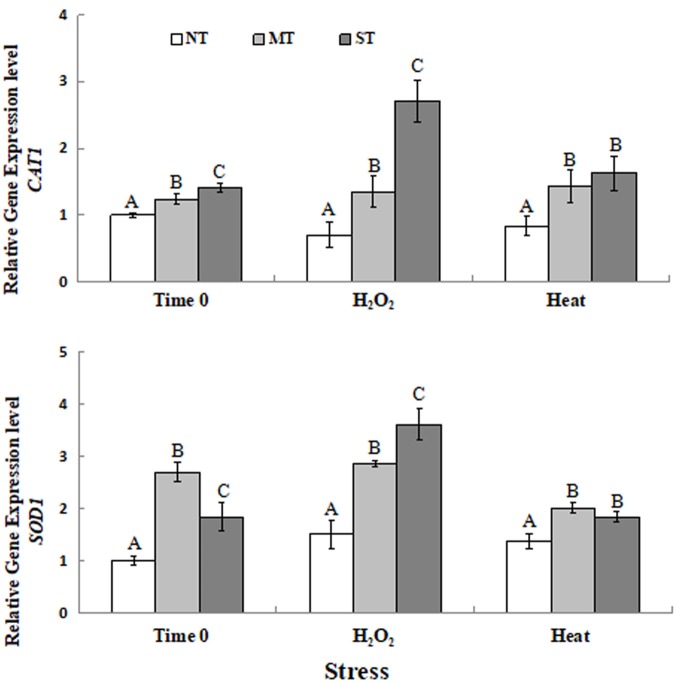
Expression of two antioxidant genes [*CAT1* **(A)** and *SOD1* **(B)**] in non-treated (NT) control, mannitol-treated (MT), and sorbitol-treated (ST) *D. hansenii* cells subjected to a subsequent oxidative (30 mM H_2_O_2_), or heat (40.5°C) stress for 30 min, at time 0 (prior to exposure to the subsequent oxidative or heat stress). Data represent the mean ± standard deviation of three independent experiments, where each experiment consisted of three biological replicates (*n* = 9). Columns with different letters are significantly different according to a Duncan’s multiple range test at *p* < 0.05.

Results of the RT-qPCR analysis on the expression of *CAT1*, *SOD1* transcripts in MT, ST, and NT cells of *D. hansenii* is presented in Figure 3. The RT-qPCR analysis of gene expression of yeast samples at time 0 (the time point after the 2-hour pretreatment but prior to exposure to the oxidative or high-temperature stress) indicated that pretreatment of yeast cells with sorbitol or mannitol upregulated the expression of *CAT1* and *SOD1* in *D. hansenii* cells. The expression of *CAT1* was significantly higher in MT and ST cells than in NT cells exposed to 30 mM H_2_O_2_ (30 min). Similarly, the expression of *CAT1* in cells exposed to 40.5°C (30 min) yeast cells was also elevated (Figure 3A). The expression of *SOD1* in pretreated cells was significantly higher than in NT cells exposed to 30 mM H_2_O_2_ (30 min). The expression of *SOD1* in cells exposed to 40.5°C (30 min) was also significantly higher in MT and ST cells than in NT cells (Figure 3B).

### Effect of Mannitol and Sorbitol Treatments on Antioxidant Enzyme Activity of *D. hansenii*

**FIGURE 4:**
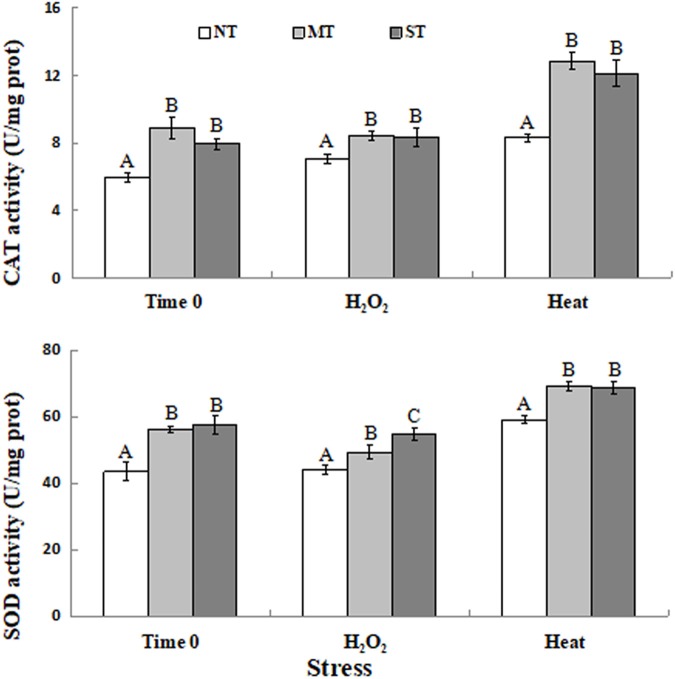
CAT **(A)** and SOD **(B)** activity in non-treated (NT) control, mannitol-treated (MT), and sorbitol-treated (ST) of *D. hansenii* cells subjected to a subsequent oxidative (30 mM H_2_O_2_), or heat (40.5°C) stress for 30 min, at time 0 (prior to exposure to the subsequent oxidative or heat stress). Data represent the mean ± standard deviation of three independent experiments, where each experiment consisted of three biological replicates (*n* = 9). Columns with different letters are significantly different according to a Duncan’s multiple range test at *p* < 0.05.

The activity of catalase and SOD activity were measured in the pretreated and control samples in response to the oxidative and high-temperature stress treatments. Results indicated that CAT and SOD activity was significantly higher in MT and ST yeast cells than in NT yeast cells (Figure 4). The activity of these antioxidant enzymes, as with the level of expression of their corresponding genes, was significantly higher in yeast cells of *D. hansenii* pretreated with mannitol or sorbitol than in untreated cells prior to and after exposure (time 0 and after 30 min) to the subsequent high-temperature and oxidative stress. CAT activity in MT and ST cells in response to exposure to the high-temperature and oxidative stress was significantly higher than in NT cells (Figure 4A). SOD activity was also significant higher in MT and ST cells exposed to the high-temperature and oxidative stress than in NT cells at all of the evaluated time points (Figure 4B).

### Intracellular Content of Mannitol and Sorbitol in *D. hansenii*

**FIGURE 5:**
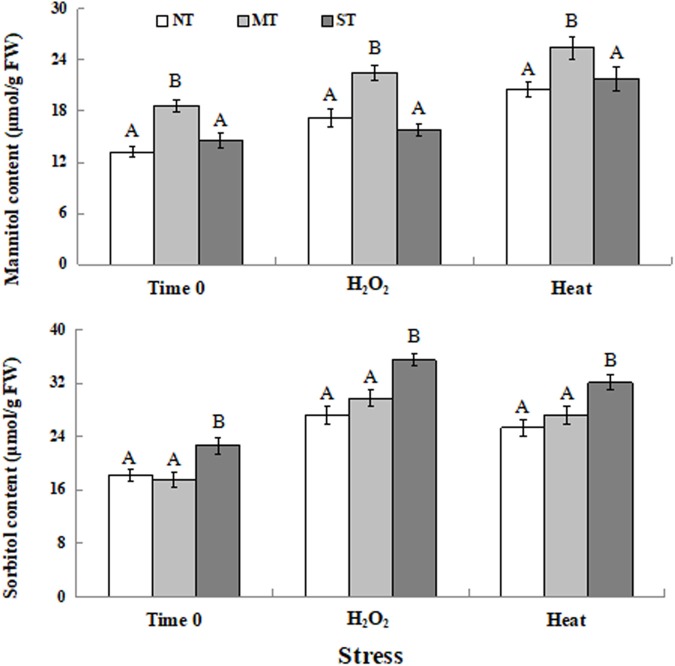
Intracellular contents of mannitol **(A)** and sorbitol **(B)** in *D. hansenii* cells subjected to a subsequent oxidative (30 mM H_2_O_2_), or heat (40.5°C) stress for 30 min, at time 0 (prior to exposure to the subsequent oxidative or heat stress). Mannitol and sorbitol concentrations represented as μmol per gram of yeast cell fresh weight (μmol/g FW). Data represent the mean ± standard deviation of three independent experiments, where each experiment consisted of three biological replicates (*n* = 9). Columns with different letters are significantly different according to a Duncan’s multiple range test at *p* < 0.05.

As indicated in Figure 5, the intracellular level of mannitol in MT cells was significantly higher than it was in ST and NT cells at time 0 (the time point after the 2-hour pretreatment but prior to exposure to the oxidative or high-temperature stress). Exposure to either a 30-minute treatment of 30 mM H_2_O_2_ or 40.5°C elevated intracellular mannitol content, with MT cells still exhibiting the highest mannitol content (Figure 5A). Similarly, ST cells had the highest intracellular sorbitol content at time 0 and under the stress conditions (Figure 5B).

### Growth of *D. hansenii* in Kiwifruit Wounds

**FIGURE 6:**
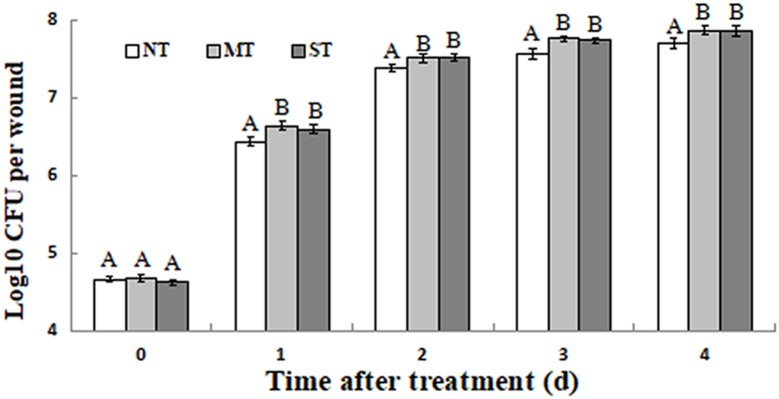
Growth dynamics of non-treated (NT) control, mannitol-treated (MT), and sorbitol-treated (ST) *D. hansenii* in kiwifruit wounds at 25°C. Fruits were wounded and inoculated with 10 μL suspension of MT or ST and NT yeast cells at 1 × 10^7^ cells/mL. Data represent the mean ± standard deviation of three independent experiments, where each experiment consisted of three biological replicates (*n* = 9). Columns with different letters are significantly different according to a Duncan’s multiple range test at *p* < 0.05.

All of the *D. hansenii* cultures (NT, MT, and ST) grew rapidly after they were administered to fruit wounds. The population of MT and ST cells, however, was significantly higher than the population of NT cells on each of 4 days following their inoculation into wounds (Figure 6). Importantly, the ability of yeast biocontrol agents to rapidly establish themselves and grow in fruit wounds once they are administered is considered a biocontrol trait that is essential for effective control.

### Biocontrol Assay of *D. hansenii* Against Postharvest Diseases of Kiwifruit Fruits

**FIGURE 7:**
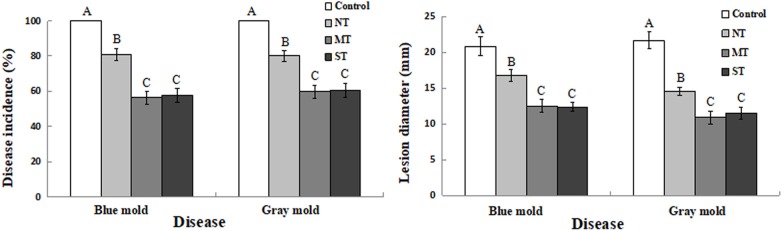
Biocontrol efficacy of *D. hansenii* against *P. expansum* and *B. cinerea* on kiwifruit stored at 25°C. Fruits were wounded inoculated with 10 μl of sterile water (control), and non-treated (NT), mannitol-treated (MT), and sorbitol-treated (ST) cell suspension of *D. hansenii* at 1 × 10^7^ cells/ml. Fruits were air-dried and then wounds were inoculated with either 10 μl of *P. expansum* or *B. cinerea* at 1 × 10^4^ spores/ml. Disease incidence **(A)** and lesion diameter **(B)** in kiwifruit were recorded after 4 days. Data represent the mean ± sd of the pooled data from three experiments (*n* = 9). Columns with different letters at each time point indicate significant differences according to a Duncan’s multiple range test at *P* < 0.05.

As shown in Figure 7, the antagonistic yeast, *D. hansenii*, significantly reduced both disease incidence and lesion diameter of both blue mold and gray mold infections of kiwifruit caused by *P. expansum* and *B. cinerea*, respectively. Notably, the incidence of both blue and gray mold decay in fruit treated with NT cultures was about 20% lower than the control group where no yeast cells were administered, which had an incidence level of 100% for both pathogens. In contrast, disease incidence in kiwifruit treated with MT or ST cell groups was reduced significantly, relative to the NT control (Figure 7A). Correspondingly, lesion diameters caused by *P. expansum* and *B. cinerea* were significantly smaller on kiwifruit fruits inoculated with MT or ST cells, compared to lesion size in kiwifruit treated with NT cells (Figure 7B). These data indicate that *D. hansenii* significantly reduced both disease incidence and lesion size for both gray mold and blue mold pathogens, however, the use of yeast cells pretreated with mannitol or sorbitol further increased the level of control beyond the level observed for non-treated (NT) cells.

## Discussion

Biocontrol agents used to manage postharvest diseases encounter a variety of abiotic stresses under packinghouse conditions, including oxidative stress, high temperature, lack of nutrients, and adverse pH. Enhancing the viability and efficacy of yeasts exposed to these stresses represents a useful strategy for increasing the performance of postharvest biocontrol agents in both packinghouses and in preharvest field applications (Teixidó et al., 2006; Wang et al., 2010; An et al., 2012). In the present study, yeast viability under oxidative and high temperature conditions was determined for yeast cultures that were pretreated with a 2 h exposure to 0.1 M mannitol (MT) or 0.1 M sorbitol (ST). Results indicated that the survival rate of MT and ST yeast determined by CFU counting was significantly higher compared to untreated (NT) yeast cells that had not been exposed to either mannitol or sorbitol (Figure 1). Several methods for enhancing stress tolerance and improving the efficacy of biological control have been previously reported. These include, stress adaptation (Liu et al., 2012; Wang et al., 2018), physiological manipulation (Abadias et al., 2001; Mokiou and Magan, 2008), and the use of exogenous anti-stress substances (Liu et al., 2011; An et al., 2012; Sui et al., 2012). Notably, viability in the present study was estimated as CFUs that only took into account viable cells with growth capacity. Some yeast cells with metabolic activity and still able to inhibit molds, but not able to multiply viable but non-culturable cells (VBNC) may have been present. These cells may exhibit a low but detectable level of metabolic activity and gene expression, as well as maintain membrane integrity, but not able to grow on the culture medium and therefore do not form a CFU. In this case, actual levels of survival could be higher than the level determined by CFUs.

Mannitol is a common metabolite in many organisms where it is synthesized in response to abiotic stress and functions as an osmoprotectant, a free radical scavenger, and a stabilizer of protein and membrane structure (including in chloroplasts, thus helping to maintain photosynthesis) (Seckin et al., 2009). Sorbitol stabilizes the native conformation of proteins, counteracting the detrimental effects of desiccation and high temperature stresses (Wolfe et al., 1998). Sugar alcohols have been reported to enhance stress tolerance and have a significant antioxidant effect in many plants (Pharr et al., 1995). *Debaryomyces hansenii* is a halotolerant yeast that produces and assimilates a wide variety of polyols (Pereira et al., 2014). In our study, both mannitol and sorbitol enhanced the viability of *D. hansenii* under oxidative stress and high temperature conditions (Figure 1). When yeasts are exposed to severe stresses, such as oxidative and heat stress, large amounts of intracellular ROS are generated that impair cell viability (Liu et al., 2012; Sui and Liu, 2014). An et al. (2012) reported that exogenous calcium improves the viability of the biocontrol yeasts, *D. hansenii* and *Pichia membranaefaciens*, under heat stress by reducing ROS accumulation and oxidative damage to cellular proteins. Liu et al. (2017) found that three ion-modified (Mg^2+^, Fe^2+^, and Zn^2+^) minimal mineral media enhanced viable biomass production and biocontrol efficacy of the biocontrol yeast *Candida diversa* by protecting cells from oxidative damage and elevating of the activity of SOD and glutathione peroxidase enzymes. In the present study, a lower percentage of cells exhibiting ROS was observed in MT and ST yeast cells (Figure 2), and lower ROS production was associated with a higher level of viability in MT and ST cells exposed to oxidative/heat stress (Figure 1). These data indicate that exogenous mannitol and sorbitol can play an important role in increasing the level of stress tolerance in yeast used as postharvest biocontrol agents.

The expression of antioxidant genes has been reported to be upregulated in yeast cells by a variety of treatments that enhance stress tolerance (Martínez-Pastor et al., 2010; Wang et al., 2018). In the current study, pretreatment of yeast cells with sorbitol or mannitol induced a higher level of expression of the genes, *CAT1* and *SOD1*, relative to untreated cells, when all three types of yeast cells (NT, MT and ST) were exposed to a subsequent oxidative stress or high temperature (Figure 3). The increase in expression was especially evident when the yeast cells were exposed to oxidative stress (30 mM H_2_O_2_). The lack of an effective antioxidant defense system leads to an accumulation of hydrogen peroxide and/or other reactive oxygen species (ROS). The enzymatic detoxification of ROS is partially dependent on the activation of antioxidant genes such as *CAT1* and *SOD1* (Deveau et al., 2010; Huang et al., 2014; Jung and Kim, 2014). In the present study, the expression of two antioxidant genes (Figure 3) was elevated in MT and ST cells, which may have contributed to an increased ability to scavenge intracellular ROS (Figure 2) in yeast cells, and thus a higher level of viability (Figure 1).

When yeast cells are exposed to an abiotic stress, a significant increase in the level of ROS is observed. Excessive ROS levels can result in oxidative damage to cellular constituents, including nucleic acids and proteins, resulting in a decreased enzyme activity in cells (Reverter-Branchat et al., 2004; Branduardi et al., 2007). In our study, CAT enzyme activity in MT and ST cells exposed to subsequent oxidative (30 mM H_2_O_2_ for 30 min) or high-temperature (40.5°C for 30 min) stress was significantly higher than in NT cells. Similarly, MT and ST yeast cells also exhibited significantly higher SOD enzyme activity compared to NT yeast cells, when the different groups of yeast cells were exposed to a subsequent oxidative or high-temperature stress (Figure 4). This pattern of enzyme activity did not exactly match levels of gene expression (Figure 3). This may be attributed to the fact that the measurement of enzyme activity was for total CAT and SOD activity, while the level of gene expression was for specific genes, *CAT1* and *SOD1*. Alternatively, post-transcriptional, post-translational, or different rates of protein turnover vs. transcript turnover may have also account for the discrepancy between levels of gene expression and enzyme activity. However, the inducive effect of MT and ST on both transcription and enzyme levels was clearly evident. Previous researches have reported that increased antioxidant enzyme activity contributes to improved abiotic stress tolerance (Collinson et al., 2002; Chi et al., 2015; Sui et al., 2015; Akshya and Sukesh, 2017; Wang et al., 2018).

Mannitol and sorbitol are reported to play important roles in the response of yeast cells to a variety of abiotic stresses (Teixidó et al., 1998; Abadias et al., 2000; Kumar and Gummadi, 2009; Hua et al., 2015). Managbanag and Torzilli (2002) reported that mannitol accumulated in cells of a yeast-like fungus, *Aureobasidium pullulans*, during exposure to heat and salt stress. Aguilera and Prieto (2001) observed that intracellular sorbitol content in *Saccharomyces cerevisiae* cells increased when cells were exposed to oxidative, heat and salt stresses. Similar increases in intracellular mannitol and sorbitol levels were observed in *D. hansenii* under oxidative and heat stress in the present study (Figure 5). Moreover, exposure to exogenous MT increased the concentration of intracellular mannitol (Figure 5A), while exposure to exogenous ST increased the intracellular level of sorbitol (Figure 5B). This indicated that *D. hansenii* cells were able to absorb a certain amount of exogenous mannitol or sorbitol. The increased levels of mannitol and sorbitol may play a crucial antioxidant function in yeast cells exposed to oxidative or heat stress, thus enabling cells to maintain a higher level of viability (Figure 1).

Rapid growth of yeast biocontrol agents in wounds and fruit surfaces is an advantage for microbial antagonists competing for nutrients and space (Liu et al., 2013). MT and ST yeast cells grew more quickly than NT cells in wounds of kiwifruit fruits over the sampled 4-day time period (Figure 6). Previous studies have also reported that pretreatment of yeast cells with different exogenous compounds can significantly enhance their biocontrol efficacy (Sui et al., 2012). In the present study, MT and ST cells reduced the incidence of blue mold and gray mold to a significantly greater degree than NT yeast (Figure 7A). The lesion diameters caused by pathogenic fungi on kiwifruit were also significantly smaller in fruits treated with MT and ST yeast cells, compared to kiwifruit treated with NT cells (Figure 7B). These results are in agreement with previous studies indicating that specific types of pretreatment of antagonistic yeast can activate their antioxidant defense system and increase their biocontrol efficacy (Chi et al., 2015; Sui et al., 2015; Wang et al., 2018).

## Conclusion

Pretreatment of *D. hansenii* yeast cells with mannitol or sorbitol increased their survival rate, antioxidant enzyme activity and gene expression, and decreased intracellular ROS level in the pretreated yeast when subsequently exposed to oxidative or high-temperature stress. The pretreatment of yeast with mannitol or sorbitol was also associated with the upregulation of intracellular mannitol or sorbitol level. The enhancement in stress tolerance increased the biocontrol efficacy of *D. hansenii* in controlling blue mold and gray mold decay on kiwifruit. While many studies on the function and use of mannitol and sorbitol have been conducted on animals and higher plants, few studies have examined their effect on biocontrol yeast. The results of our study may have practical implications for the use of mannitol and sorbitol to improve ability of yeast to control postharvest diseases of fruits. However, the effects of mannitol and sorbitol treatment on the tolerance of biocontrol yeasts to additional stresses, like osmotic and cell wall stresses, need to be further investigated.

## Data Availability Statement

The datasets generated for this study are available on request to the corresponding author.

## Author Contributions

YW and YS conceived and designed the experiments. All authors performed the experiments and analyzed the data, read and approved the final manuscript. YS drafted the manuscript.

## Conflict of Interest

The authors declare that the research was conducted in the absence of any commercial or financial relationships that could be construed as a potential conflict of interest.
